# Self-organization of unimolecular micelles in beam stream for functional mesoporous metal oxide nanofibers

**DOI:** 10.1016/j.fmre.2021.12.002

**Published:** 2021-12-11

**Authors:** Chongfei Gu, Xiaoshan Fan, Guihua Zhu, Yuchi Fan, Haifeng Wang, Tao Zhao, Qi Xiao, Yuan Fang, Xiaopeng Li, Wan Jiang, Lianjun Wang, Pengpeng Qiu, Wei Luo

**Affiliations:** State Key Laboratory for Modification of Chemical Fibers and Polymer Materials, College of Materials Science and Engineering, Institute of Functional Materials, Donghua University, Shanghai 201620, China

**Keywords:** Unimolecular micelles, Self-assembly, Electrospinning, Mesoporous metal oxides, Nanofibers

## Abstract

The use of linear amphiphilic block copolymers as templates is an important method for the preparation of mesoporous materials. However, the obtained assemblies are usually sensitive to synthetic conditions, which impedes the preparation of such mesoporous materials in certain environments. Herein, we report a universal strategy applying an amphiphilic multi-arm triblock copolymer in the preparation of mesoporous metal oxide nanofibers (NFs) using one metal oxide (TiO_2_, ZrO_2_, WO_3_, CeO_2_), or two (TiO_2_/WO_3_, TiO_2_/ZrO_2_, TiO_2_/CeO_2_) and three (TiO_2_/WO_3_/CuO) metal oxides as composites. The template consists of modified β-cyclodextrin as the center of the macromolecule which is attached sequentially to a block of polystyrene, poly(acrylic acid), and poly(ethylene oxide). Under electrospinning conditions, stable unimolecular micelles are formed and effectively co-assemble with metal ions to form fibrous nanostructures. As indicated by various characterization methods, the synthesized TiO_2_ and its derived composite NFs maintain a straight and continuous fibrous structure after calcination, and TiO_2_ NFs exhibit uniform mesopores of 10.8 nm in diameter and a large Brunauer-Emmett-Teller surface area of 143.3 m^2^ g^−1^. Benefiting from the characteristic structure, still present after modification, Pt-decorated mesoporous TiO_2_ NFs display excellent ability in the visible-light photocatalytic degradation of tetracycline, which is superior to the commercial P25 catalyst. This study reveals a promising strategy for the preparation of fibrous mesoporous metal oxides.

## Graphical abstract

**Figure:**
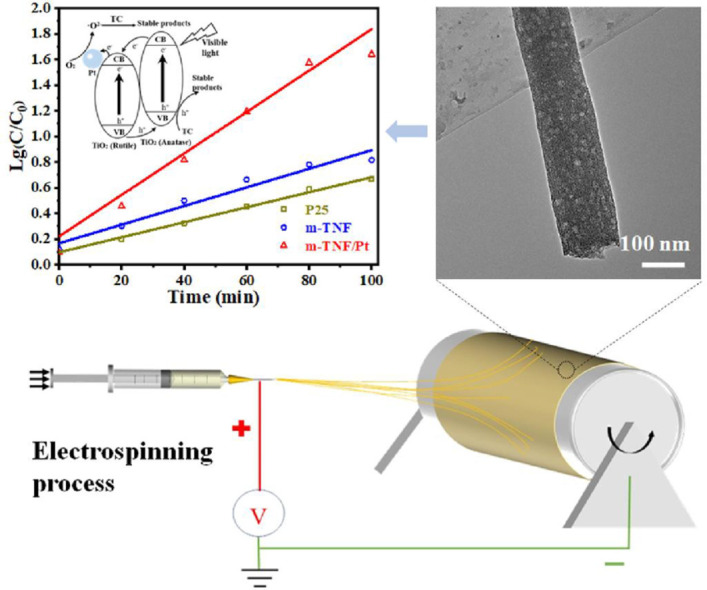

## 1. Introduction

Recently, mesoporous metal oxides (MOs) with diverse morphologies have gained increasing attention in many application fields such as catalysis [1], [2], [3], [4], energy conversion and storage [5], [6], [7], [8], sensing [9], [10], [11], [12], and dye-sensitized solar cells [13], [14], [15], [16]. Particularly, one-dimensional (1D) nanofibers (NFs) are of great importance because of their large aspect ratio, which can provide short and effective mass and electron transfer pathways [17], [18], [19]. However, 1D MOs composed of both mesoporous structure and fibrous morphology have rarely been successfully manufactured due to limitations in the methodologies of material synthesis. Firstly, linear block copolymers form assembled structures (e.g., micelles, vesicles, and lamellas) in solution when present in concentrations above their critical micelle concentration [20], [21], [22], [23]. These assemblies are dynamically stable, and their properties are largely dependent on the experimental conditions (temperature, pH, concentration, and solvent type) [24]. Therefore, the precise tuning of the experimental conditions to achieve micellation of the template and anisotropic growth of the micelles along 1D direction is essential but difficult, which constraints the synthetic scope of MOs. Secondly, the used metal ions typically exhibit a fast hydrolysis and condensation rate [25], which hinders effective interaction with the template to form uniform composite micelles. The third obstacle is related to the feasibility of forming fibrous structures, which requires building an appropriate hard template for heterogeneous growth and simultaneously controlling the assembly of molecular building blocks precisely [26]. Therefore, the development of an effective synthetic strategy for mesoporous MO NFs is widely pursued, and numerous high-value targets still remain inaccessible.

Herein, we demonstrate a convenient and universal strategy to prepare mesoporous MO NFs based on a novel multi-arm amphiphilic macromolecule consisting of a core and two shells as the pore-forming agent and on the application of an electrospinning technique. Compared with conventional micelles, the multi-arm core–double-shell macromolecules consist of hydrophilic and hydrophobic blocks covalently bonded to a dendritic core and form unimolecular micelles. The unimolecular structure of the amphiphile avoids the complex dynamic assembly that dominates the synthesis of most linear amphiphiles, and the curvature constraints of this architecture generate unconnected spherical pores in high volume fractions [27], [28], [29], [30]. Its utilization as a porogenic matrix is regarded as a strategy between soft-templating and hard-templating because it combines the high stability of hard templates and the low evolution degree of soft templates. Mesoporous TiO_2_ NFs (m-TNFs) and TiO_2_-based composite NFs are chosen as examples to demonstrate the general applicability of this strategy. The prepared m-TNFs possess well-defined 1D orientation with large mesopores (10.8 nm) and a large surface area (143.3 m^2^ g^−1^). More importantly, this strategy can be easily extended to synthesize other MO NFs (e.g., CeO_2_, ZrO_2_, and WO_3_), and even their composite fibers. Due to the large aspect ratio and high specific surface area, the 1D mesoporous NFs can not only offer effective paths for electron transfer but also provide abundant interfaces for interactions between pollutant molecules and catalysts. Therefore, Pt-decorated m-TNFs (m-TNF/Pt) were synthesized in this study and demonstrated superior photocatalytic degradation ability (81.4%) of tetracycline (TC) as well as excellent recyclability. This study presents a general strategy for the synthesis of mesoporous MO and MO-composite NFs with promising applications in a wide range of fields.

## 2. Results and discussion

An amphiphilic β-cyclodextrin (β-CD)-based polymer containing three sequential blocks of polystyrene (PS), poly(acrylic acid) (PAA), and poly(ethylene oxide) (PEO) was prepared in this work. This multi-arm β-CD-PS-*b*-PAA-*b*-PEO triblock copolymer was synthesized using α-bromo-isobutyryl-modified β-CD (β-CD-Br) as initiator for an atom transfer radical polymerization (ATRP) reaction with styrene, followed by a second ATRP reaction with *t*-butyl acrylate, a one-step click reaction with poly(ethylene oxide) methyl ether, and a hydrolysis process (Fig. S1). To verify the quality of the template, ^1^H NMR spectra were recorded (Fig. S2). In the ^1^H NMR spectrum of β-CD-Br, the apparent singlet with a chemical shift at 1.8-2.2 ppm was attributed to the methyl protons in the bromoisobutyryl groups, while the peaks in the range of 3.5-5.5 ppm were assigned to the remaining protons of β-CD-Br. According to the ratio of the two peak areas, the conversion rate of the hydroxyl groups was calculated to be 100%, proving that all hydroxyl groups of β-CD completely reacted to the corresponding bromoisobutyrate esters [31, 32]. After the first ATRP reaction, the PS block was successfully grafted on the β-CD-Br initiator, which was verified by the appearance of the characteristic peak for the protons of the PS benzene rings in the ^1^H NMR spectrum of β-CD-PS (Fig. S2a, b) [31]. For the products in the following synthetic steps, the characteristic peaks at 1.44 ppm (peak g) and 3.58 ppm (peak h) were assigned to the methyl protons of the *t*-butyl groups of the poly(*t*-butyl acrylate) (P*t*BA) block and the ethyl protons of the PEO block, respectively, suggesting that the P*t*BA and PEO blocks were also successfully added (Fig. S2c, d) [33, 34]. The relative molecular weights of the PS and P*t*BA segment were calculated as 3450 and 5100 g mol^−1^, respectively. Thus, the chemical formula of the multi-arm triblock copolymer was determined as β-CD-(PS_33_-*b*-P*t*BA_40_-*b*-PEO_114_)_21_. The polymer dispersity index (PDI) derived from the gel permeation chromatography result was 1.12, indicating a narrow molecular weight distribution. After hydrolyzing P*t*BA by trifluoromethanesulfonic acid, the PDI changed to 1.33, still maintaining a narrow molecular weight distribution, which is conducive to the preparation of mesoporous fibers of uniform pore size.

**Fig. 1:**
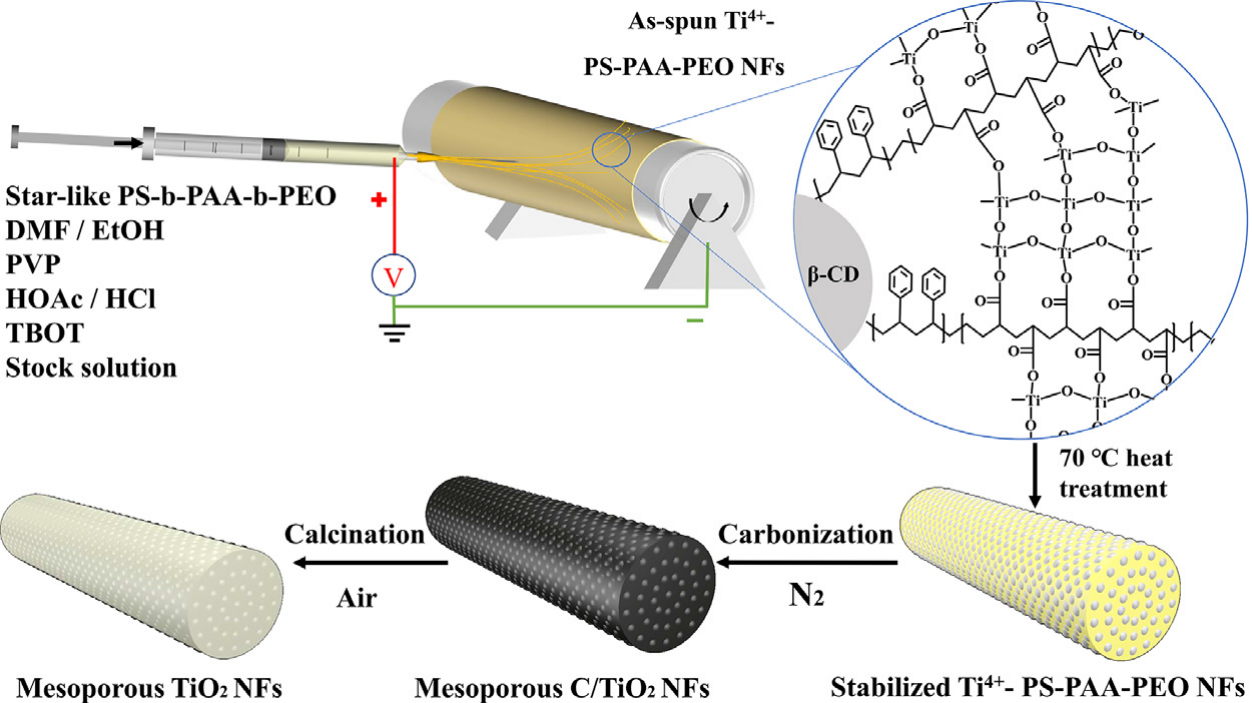
**Schematic illustration for preparing m-TNF through the self-organization of unimolecular micelles in the charged beam generated by the electrospinning**.

**Fig. 2:**
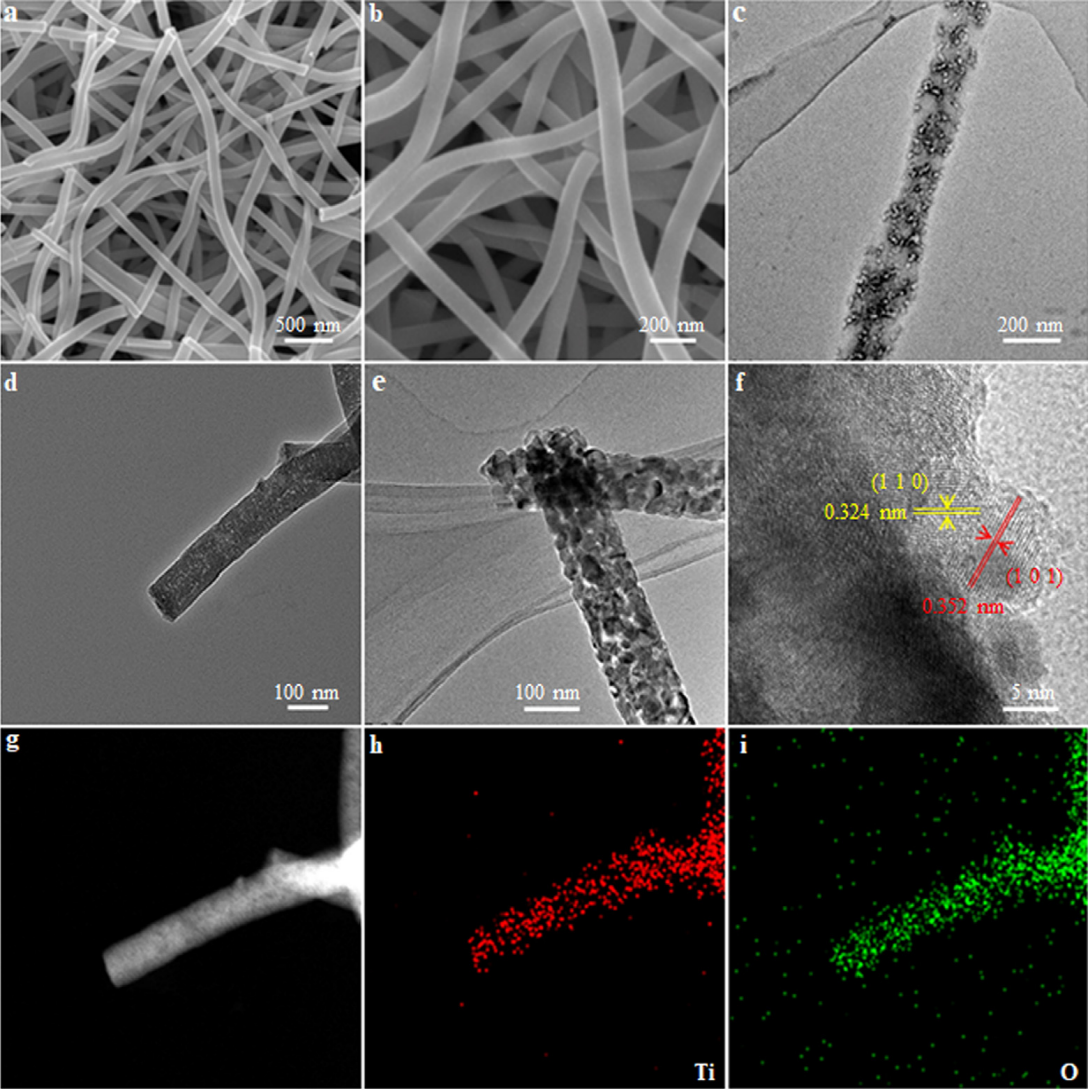
**Morphology characterization of mesoporous TiO_**2**_ nanofibers**. (a, b) SEM images of m-TNFs. TEM images of (c) as-spun Ti^4+^-template fibers, (d) mesoporous C/TiO_2_ NFs, and (e) m-TNFs. (f) HRTEM images of m-TNFs. STEM image of m-TNFs (g) and EDX maps of Ti (h) and O (i).

**Fig. 3:**
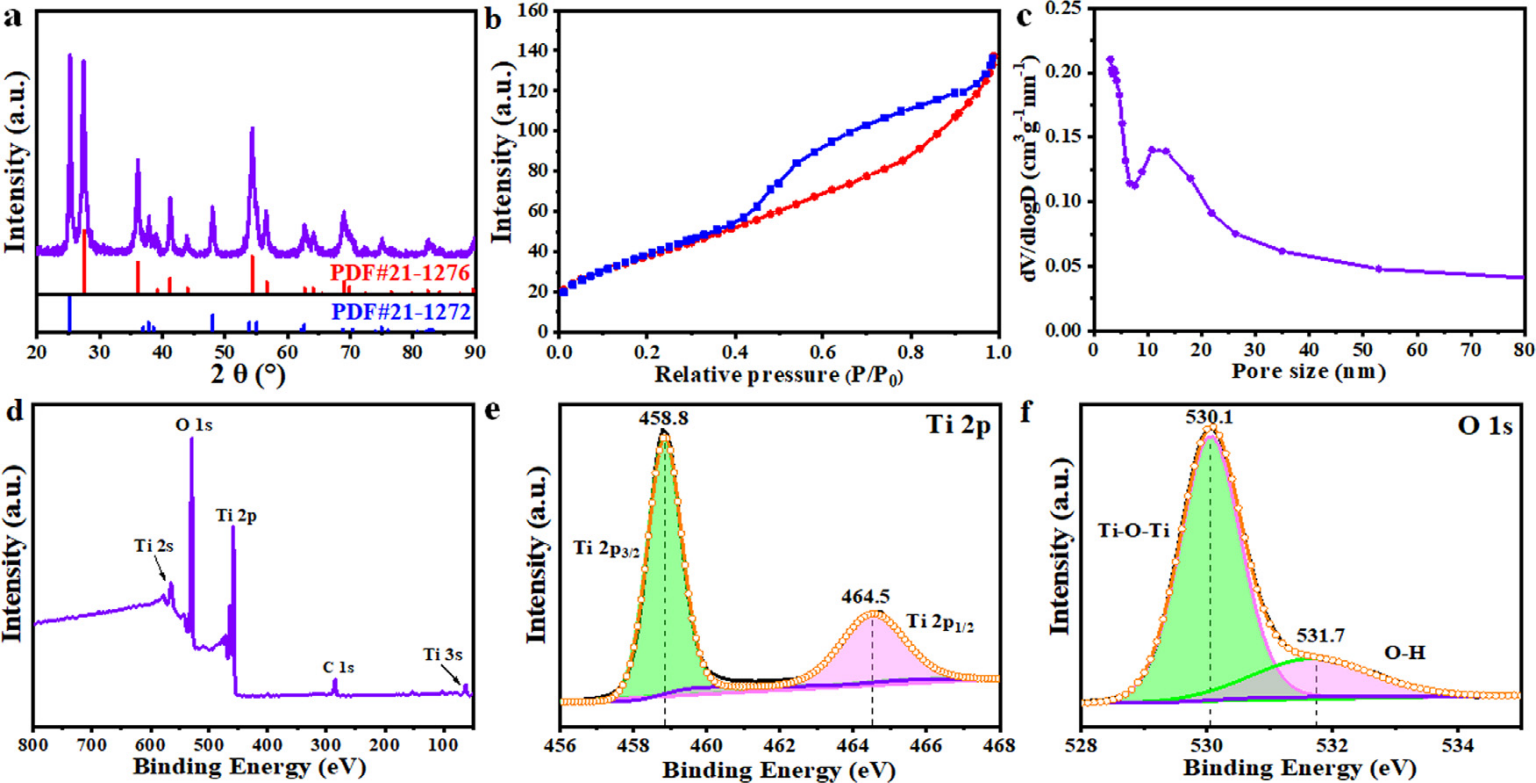
**Composition and structure characterization of m-TNF**. (a) XRD pattern of m-TNF, (b) the nitrogen adsorption-desorption isotherms of m-TNF and (c) the corresponding pore-size distribution curve, (d) XPS spectra of m-TNF, high-resolution (e) Ti 2p and (f) O 1s XPS spectra of m-TNF.

As an example, mesoporous TiO_2_ NFs were constructed in a charged beam using polyvinyl pyrrolidone (PVP) as a thickening agent and DMF as the solvent through the self-organization of micelles formed by the multi-arm macromolecules and titanium(IV) butoxide as the titanium source, followed by a continuous calcination process in first nitrogen atmosphere and then air atmosphere (Fig. 1). SEM images of as-spun Ti^4+^-PS-PAA-PEO NFs show a continuous, long, and straight morphology with a fiber diameter of about 115 nm (Fig. 2a, b). TEM images further demonstrate that the as-spun NFs exhibit a continuous and straight structure, which are stacked by the composite micelles (Fig. 2c). After calcining in nitrogen atmosphere, C/TiO_2_ NFs were formed (Fig. 2d), maintaining the fibrous structure with large mesopores (10.3 ± 0.5 nm). After removing the carbon framework by calcining in air, pure mesoporous TiO_2_ NFs were obtained **(**Fig. 2e**)**. The high-resolution TEM (HRTEM) image revealed that the pore walls of the m-TNFs were composed of highly crystalline TiO_2_ with two different lattice fringes (0.352 and 0.324 nm), attributed to the (1 0 1) plane of anatase and the (1 1 0) plane of rutile, respectively (Fig. 2f). The formation of the two phases was also proved by X-ray diffraction (XRD) analysis (Fig. 3a). The scanning TEM (STEM) image further indicated that m-TNFs possessed uniform mesopores (Fig. 2g). In addition, their corresponding elemental mappings obtained by energy-dispersive X-ray spectroscopy (EDX) revealed the uniform distribution of Ti and O atoms throughout the entire material (Fig. 2h, i).

The nitrogen adsorption-desorption isotherm of m-TNFs depicts a typical type IV curve with an H_1_ hysteresis loop, confirming that the materials possessed uniform mesopores (Fig. 3b). The BET surface area and pore volume of m-TNFs were determined as 143.3 m^2^ g^−1^ and 0.21 cm^3^ g^−1^, respectively. The pore size distribution (PSD) derived from the adsorption branch using the BJH model displays a narrow peak centered at 10.8 nm (Fig. 3c**)**, revealing the formation of a mesoporous structure in large domains. As a comparison, the sample synthesized without the multi-arm template was also examined. In the PSD curve of TiO_2_ NFs without the template (Fig. S3), only one peak attributed to the phase separation of PVP was obtained, suggesting that the multi-arm template plays a critical role in the formation of large mesopores in the NFs. The TEM image of the NFs shows a clear nonporous structure in NF direction (Fig. S4). Two peaks related to Ti 2p and O 1s were detected in the X-ray photoelectron spectroscopy (XPS) spectrum of m-TNFs, corroborating the formation of TiO_2_ (Fig. 3d). The Ti 2p XPS spectrum of m-TNF features two peaks at 458.8 and 464.5 eV, which were assigned to Ti^4+^ 2p3/2 and Ti^4+^ 2p1/2, respectively (Fig. 3e) [35]. The high-resolution O 1s spectrum was deconvoluted into two peaks with binding energies of 530.0 and 531.7 eV (Fig. 3f), which were attributed to the Ti-O-Ti bond in the structure and the O-H bond on the material surface, respectively [36].

**Fig. 4:**
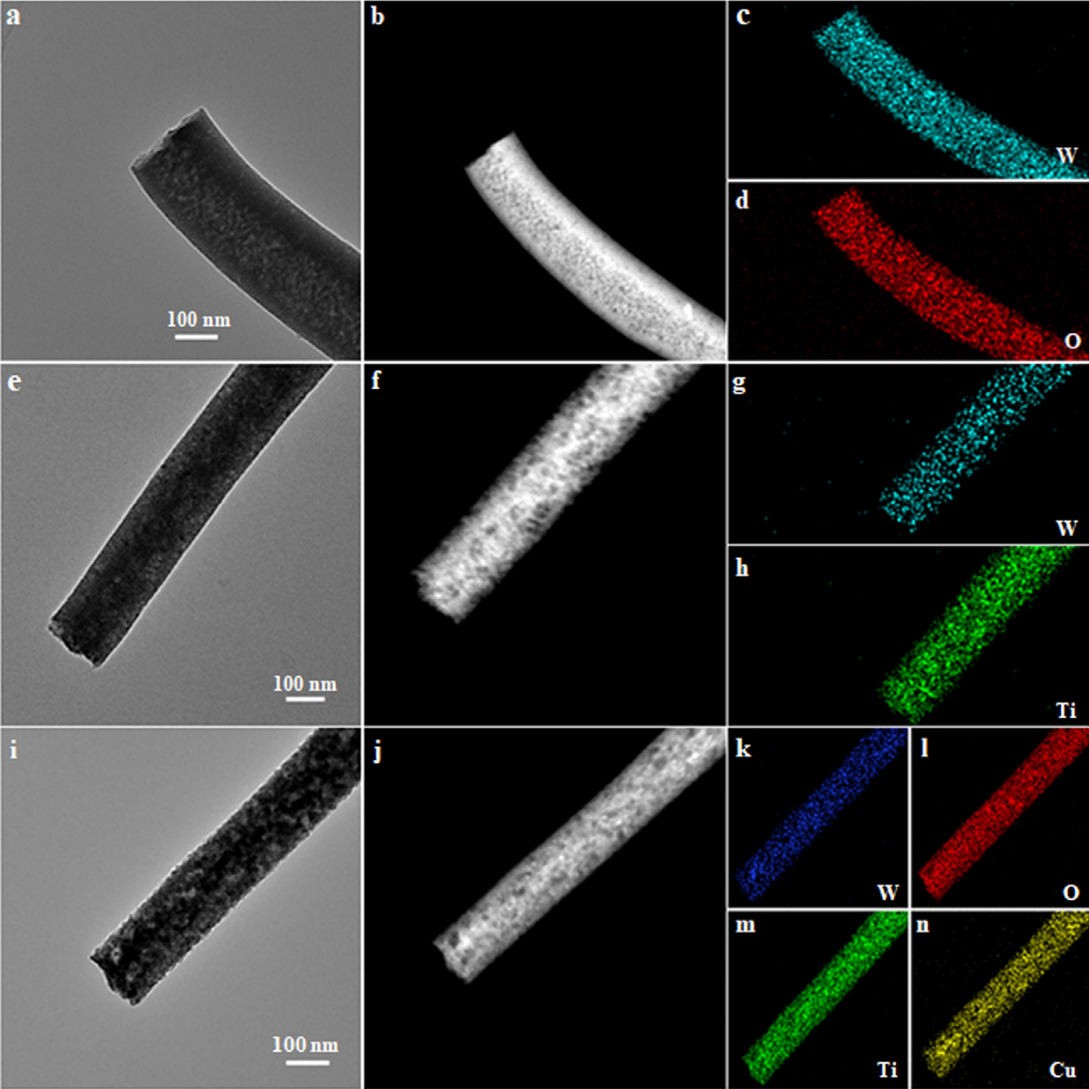
**Morphology characterization and element analysis of composite nanofibers**. (a) TEM and (b) STEM images of mesoporous WO_3_ NFs and the EDX maps of (c) W and (d) O. (e) TEM and (f) STEM images of mesoporous TiO_2_/WO_3_ NFs and the corresponding EDX maps of (g) W and (h) Ti. (i) TEM and (j) STEM images of mesoporous TiO_2_/WO_3_/CuO NFs and the corresponding EDX maps of (k) W, (l) O, (m) Ti, and (n) Cu.

Since this strategy is very general, it is suitable for the fabrication of other MOs (e.g., WO_3_, ZrO_2_, and CeO_2_). SEM images of these MOs (Fig. S5**)** show a comparable continuous, long, and straight NF structure. TEM images of these MOs also indicate a definite NF with a large number of mesopores like that of m-TNFs. The STEM images (Fig. S6a, e) further prove the presence of mesopores in the entire framework of the NFs. Their corresponding elemental mappings show a uniform distribution of the metal atoms throughout the entire NFs (Figs. 4a-d and S6). No additional diffraction peaks were detected in the XRD patterns, suggesting a high purity of crystalline WO_3_, ZrO_2_ and CeO_2_ NFs (Fig. S7). More importantly, this strategy can also be extended to prepare composite NFs of bi-MOs (TiO_2_/WO_3_, TiO_2_/ZrO_2_, TiO_2_/CeO_2_) and tri-MOs (TiO_2_/WO_3_/CuO), which also possessed a straight fibrous structure with uniform mesopores. The corresponding elemental maps of W, Zr, Ce, Ti, and O further confirmed that these elements were evenly distributed in the bi-MO NFs to construct a fibrous structure (Figs. 4e-h and S8, S9). The uniform distribution of these elements proved that the TiO_2_ and WO_3_ phase are in contact and form an interphase without forming some mixed metal oxides, which is greatly conducive to the formation of heterojunctions. Peaks related to Ti 2p, Ce 3d, W 4f, Zr 3d, and O 1s were detected in the XPS spectra (Figs. S10-S12). Compared with the Ti 2p XPS spectrum of pure m-TNFs, the peaks related to Ti 2p1/2 and Ti 2p3/2 were shifted to lower binding energy, further demonstrating the formation of a heterostructure [37]. The TiO_2_/WO_3_/CuO tri-MOs NFs were also successfully formed as proved by TEM and STEM observations and the elemental maps of the material (Fig. 4i-n). All these results verify that the construction of mesoporous MOs in the charged beam by this unimolecular micelle assembly strategy is universal and feasible.

**Table 1:** **The kinetic constant for photocatalytic degradation of TC over P25, m-TNF, and m-TNF/Pt when pseudo first-ordered reaction was assumed**.

Catalysts	Rate constants (k, min^−1^)	Correlation coefficient (R^2^)
P25	6.09 × 10^−3^	0.9944
m-TNF	8.42 × 10^−3^	0.9892
m-TNF/Pt	1.91 × 10^−2^	0.9788

From the above results, it is proposed that using a charged beam in the synthesis process causes a self-organization process of unimolecular micelles to construct mesoporous MOs NFs. First, the multi-arm β-CD-PS-*b*-PAA-*b*-PEO template was dissolved in DMF to form spherical micelles, in which β-CD functions as the block center, hydrophobic PS blocks as the core, PAA blocks as the adjacent shell, and PEO blocks as the corona. TEM images clearly show that the amphiphilic β-CD-PS-*b*-PAA-*b*-PEO formed spherical unimolecular micelles with an average diameter of 12.0 ± 3.0 nm (Fig. S13a) in good agreement with the results of dynamic light scattering measurements (11.7 nm, Fig. S13b). Note that the carboxylic acid groups on PAA form negatively charged carboxylate groups in solution (zeta potential = -34.2 mV), which is beneficial for the interaction with the positively charged metal precursor and the reason why a number of MOs can be synthesized by this strategy. After high voltage is applied between the needle and the collector, the electrostatic force will deform the solution at the tip of the needle tube and produce a hanging cone-shaped droplet at the nozzle. When the charge repulsion of the droplet exceeds its surface tension, a jet is ejected from the surface of the droplet at high speed. These jets are stretched at high speed by the electric field force, and the mixed solvent rapidly evaporates within a short distance. Due to their strong stability, the unimolecular micelles can be retained under these harsh conditions. With the complete evaporation of the mixed solvent, as-spun inorganic-organic NFs are obtained after the jet is deposited on the receiver plate [38, 39]. Subsequently, calcination of Ti^4+^@β-CD-PS-*b*-PAA-*b*-PEO NFs in nitrogen atmosphere results in the carbonization of β-CD-PS-*b*-PAA-*b*-PEO and PVP to form carbon-supported TiO_2_ NFs, showing a long and continuous fibrous structure with a diameter of about 115 nm.

**Fig. 5:**
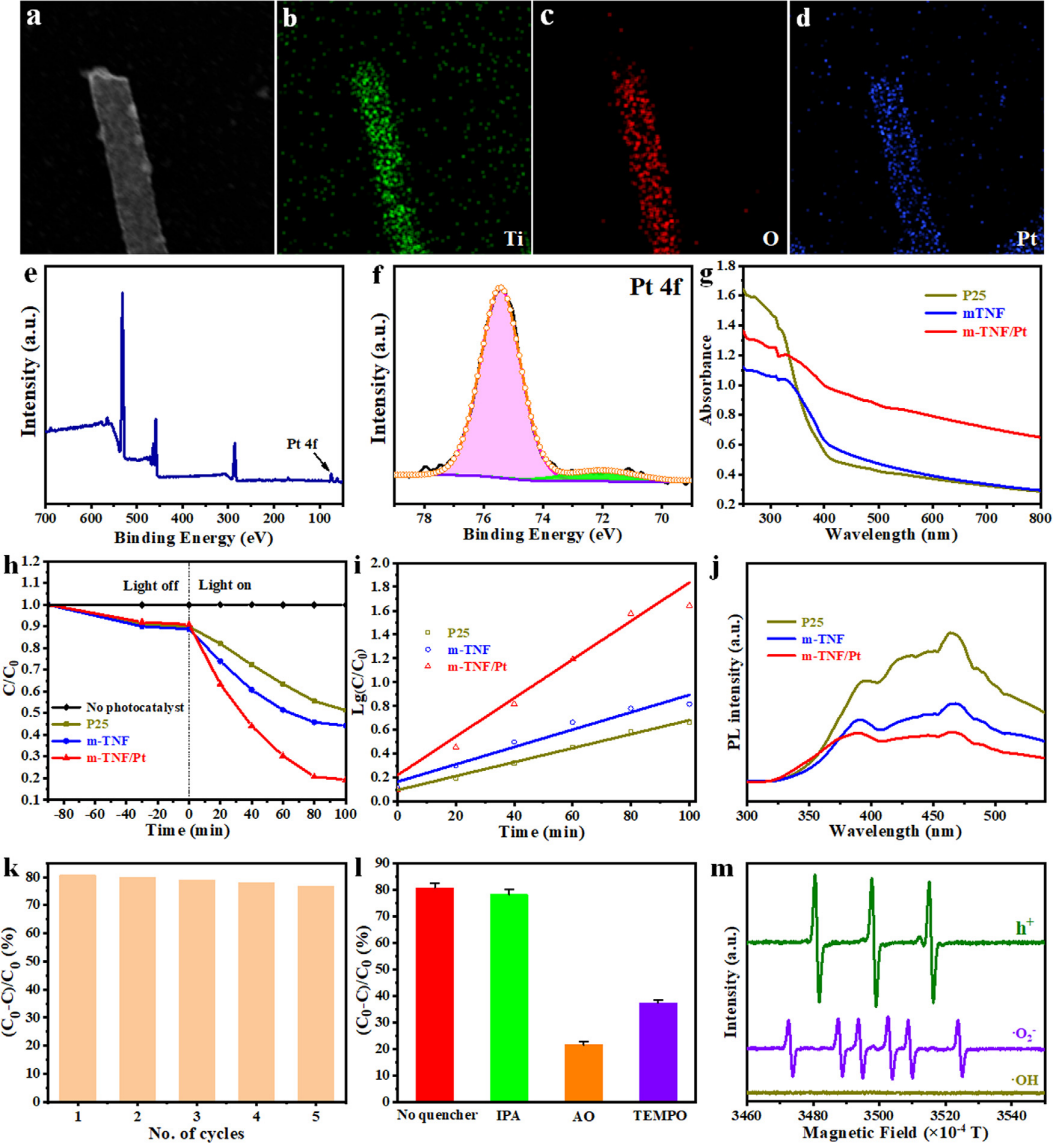
**Composition characterization of m-TNF/Pt and a series of optical performance tests**. (a) STEM image of m-TNF/Pt and the corresponding EDX maps of (b) Ti, (c) O, and (d) Pt. (e) XPS spectrum of m-TNF/Pt and (f) high-resolution Pt 4f XPS spectrum of m-TNF/Pt. (g) UV–vis absorption spectra of P25, m-TNF, and m-TNF/Pt. Visible-light photocatalytic degradation of TC over P25, m-TNF, and m-TNF/Pt: (h) removal efficiency, (i) pseudo-first-order reaction kinetics, (j) PL spectra, (k) stability test, (l) scavenging test, and (m) ESR signals

Encouraged by the unique mesoporous structure and NF morphology, m-TNFs decorated with precious metal Pt nanoparticles (NPs) were fabricated as a photocatalyst for the degradation of TC. The STEM image and corresponding EDX maps show that Pt NPs were homogeneously distributed on the surface of m-TNF (Fig. 5a-d). In addition, the detection of the Pt peak in the XPS spectrum of m-TNFs/Pt further indicated that Pt NPs were successfully loaded on m-TNFs (Fig. 5e, f). In contrast to the commercial TiO_2_ photocatalyst P25 and m-TNFs, the UV-vis absorption spectra (Fig. 5g) of m-TNFs/Pt reveal an absorption edge in the visible-light region, which is attributed to Pt sensitization. Therefore, visible-light irradiation (λ > 420 nm) was applied to excite P25, m-TNF, and m-TNF/Pt to examine their TC degradation efficiency. In the absence of photocatalyst, the visible-light photocatalytic degradation rate of TC is negligible within 100 min. In contrast, 49.6%, 56.2% and 81.4% of TC were removed in the presence of P25, m-TNF, and m-TNF/Pt, respectively (Fig. 5h). The kinetic constant of TC degradation for m-TNF/Pt (1.91 × 10^−2^) is 3.1 and 1.5 times larger than those of m-TNF (8.42 × 10^−3^) and P25 (6.09 × 10^−3^), respectively (Fig. 5i and Table 1). The higher reaction rate can be attributed to an enhanced visible-light absorption and reduced electron-hole recombination rate after Pt doping, which was proved by measuring the photoluminescence (PL) spectrum of the samples (Fig. 5j). A low PL intensity indicates a high separation efficiency of electron-hole pairs. The reduced PL intensity after immobilizing Pt suggests an improvement in the separation efficiency of the photogenerated charges. In addition, the photocatalytic stability of m-TNF/Pt for TC degradation was also investigated. No apparent decline in performance was observed after five cycles, revealing the excellent stability of m-TNF/Pt for the degradation of TC (Fig. 5k).

To gain insight into the reaction mechanism, several trapping experiments were conducted. Ammonium oxalate (AO), isopropanol (IPA), and tetramethylpiperidine oxide (TEMPO) were used as quenchers for electron hole (h^+^), hydroxyl radical (∙OH), and superoxide radical (⋅O_2_^−^), respectively [40]. The injection of TEMPO and AO significantly reduced the TC degradation kinetics, indicating that h^+^ and ⋅O_2_^−^ contribute significantly to the degradation process. The addition of IPA did not lead to a significant decrease in TC degradation, suggesting that •OH is not the dominant species (Fig. 5l). Electron spin resonance spectroscopy (ESR) results (Fig. 5m) only show the characteristic peaks for h^+^ and ⋅O_2_^−^, further verifying that h^+^ and ⋅O_2_^−^ were the dominant species in the system. This is reasonable because TC exhibits a smaller oxidation potential (2.0 V) [41] than H_2_O (2.72 V) [42], which can directly react with h^+^ instead of generating ∙OH.

## 3. Conclusion

We have reported a universal strategy for synthesizing 1D MO NFs with large mesopores through assembling unimolecular micelles in a charged beam generated by an electrospinning technique. Due to the high stability and negatively charged property of the micelles, this approach offers great feasibility for the synthesis of various MO NFs (e.g., TiO_2_, ZrO_2_, WO_3_, and CeO_2_) and their composite NFs (e.g., TiO_2_/WO_3_, TiO_2_/ZrO_2_, and TiO_2_/CeO_2_). The prepared m-TNF/Pt material was evaluated as a photocatalyst and demonstrated an excellent visible-light active degradation performance for TC, including high degradation efficiency (81.4%) and excellent recyclability. The overall performance is better than those of photocatalytic materials assessed in most previous literature due to the integrated effect of the unique mesoporous structure, 1D NF morphology, and Pt doping. This research provides an alternative for the design and synthesis of multi-component composite and functional MO NFs with great application potential in various fields.

## Declaration of Competing Interest

The authors declare that they have no conflicts of interest in this work.

## Appendix Supplementary materials

Image, application 1
