# Supplementary material for: Self-organization of unimolecular micelles in beam stream for functional mesoporous metal oxide nanofibers
Source: Fundam Res. 2021 Dec 11;2(5):776–82. doi: 10.1016/j.fmre.2021.12.002 (PMC11197481; doi:10.1016/j.fmre.2021.12.002)
Supplement: Supplementary file 1 [file mmc1.docx]

Supporting information

**Self-organization of Unimolecular Micelles in Beam Stream for Functional Mesoporous Metal Oxide Nanofibers**

Chongfei Gu, Xiaoshan Fan, Guihua Zhu, Yuchi Fan, Haifeng Wang, Tao Zhao, Qi Xiao, Yuan Fang, Xiaopeng Li, Wan Jiang, Lianjun Wang, Pengpeng Qiu* and Wei Luo*

State Key Laboratory for Modification of Chemical Fibers and Polymer Materials, College of Materials Science and Engineering, Institute of Functional Materials, Donghua University, Shanghai 201620, China.

E-mail: qiupengpeng@dhu.edu.cn (P. Qiu), wluo@dhu.edu.cn (W. Luo).

**EXPERIMENTAL SECTION**

**Synthesis of multi-arm PS-PAA-PEO**

**(a)** ***Synthesis of 21-Br-β-CD.*** *β*-cyclodextrin (*β*-CD, 1.364 g, 1.2 mmol, vacuum-dried at 80 ℃ over calcium oxide overnight immediately prior to use) was dissolved in 12 mL anhydrous 1-methyl-2-pyrrolidione (NMP) and cooled to 0 ℃. 2-Bromoisobutyryl bromide (11.6 mL, 50.4 mmol) was then added dropwise to the *β*-CD solution with magnetic stirring. The reaction temperature was maintained at 0 ℃ for 2 h and then slowly increased to ambient temperature, after which the reaction was allowed to continue for 22 h. The brown solution obtained was diluted with 60 mL dichloromethane, and then washed sequentially with saturated NaHCO_3_ aqueous solution (3×200 mL) and DI water (3×200 mL). The organic layer obtained was concentrated in a rotary evaporator, and then crystallized in cold n-hexane to produce a white precipitate.

The conversion rate of hydroxyl groups can be calculated with the following formula. [1]

$E_{T}=\frac{A_{n}}{18A_{m}}\times100\%$

Where *E_T_* means the conversion rate of -OH groups on *β*-CD, *A_n_* and *A_m_* are the integral area of the methyl protons of 21Br-*β*-CD and the integral area of the protons (H_m_), respectively. The calculated value of *E_T_* is 100%, which can prove that the -OH on *β*-CD is completely replaced by bromoisobutyryl groups.

**(b)** ***Synthesis of multi-arm β-CD-PS by ATRP using 21Br-β-CD as macroinitiator.*** Polymerization of styrene was performed using 21Br-*β*-CD as a macroinitiator possessing 21 ATRP initiation sites. An ampule charged with CuBr (0.0707 g), PMDETA (0.1707 g), 21Br-*β*-CD (0.1 g), styrene (40 mL), was degassed by three freeze-thaw cycles in liquid N_2_, then sealed and placed in an oil bath at 60 ℃. The ampule was taken out from the oil bath and dipped in liquid N_2_ at different desired times to terminate the polymerization. The solution was then diluted with tetrahydrofuran (THF) and passed through a neutral alumina column to remove the catalyst, and precipitated with an excess of methanol. After filtration, the final product was purified by dissolution-precipitation twice with THF and methanol and dried at 40 ℃ in vacuum for 2 days to yield 21-arm macromolecule, denoted as *β*-CD-PS.

**(c)** ***Synthesis of multi-arm β-CD-PS-b-PtBA by ATRP using multi-arm β-CD-PS as macroinitiator.*** All polymerizations were performed in an ampule. The reaction mixtures (*tert*-butyl acrylate (*t*BA): *β*-CD-PS (i.e., Br in PS macroinitiator): copper bromide: PMDETA = 800:1:1:2; molar ratio) in methyl ethyl ketone (1g *t*BA in 1 mL solvent) were degassed by three freeze-pump-thaw cycles in liquid N_2_ and then placed in a thermostated oil bath maintained at 90 ℃ prior to polymerization. After a desired polymerization time, the mixture was dipped in liquid N_2_ to terminate the reaction. The mixture was then diluted with THF and passed through a column of neutral alumina to remove the copper salt. The polymers were precipitated in the mixed solvents of methanol/water (v/v =1/1), filtered, and dried under vacuum to yield 21-arm diblock copolymer, denoted as *β*-CD-PS-*b*-P*t*BA.

The relative molecular mass of each block on a branch can be calculated with the following formulas. [1]

$$M_{n,PS}=\frac{{A_{d}}/5}{{A_{a}}/6}\times104.15$$

$$M_{n,PtBA}=\frac{{A_{g}}/9}{{A_{a}}/6}\times128.17$$

Where *M_n,PS_* and *M_n,PtBA_* is the M_n_ of each PS and P*t*BA arm, *A_d_*, *A_g_* and *A_a_* are the integral area of phenyl protons on the PS block, the integral area of methyl protons in *tert*-butyl group of P*t*BA chains and the integral area of methyl protons at the α-end of the block arm, respectively, and 104.15 and 128.17 is the molecular weight of St and *t*BA monomer.

**(d)** ***Synthesis of multi-arm β-CD-PS-b-PtBA-b-PEO by click reaction.*** The *β*-CD-PS-*b*-P*t*BA (2.0 g) was dissolved in DMF (15 mL), and sodium azide (Br in *β*-CD-PS-*b*-P*t*BA: sodium azide =1: 10; molar ratio) was added to the solution. The reaction mixture was stirred for 24 h at room temperature. Dichloromethane (25.0 mL) was added to the mixture. The mixture was then washed with distilled water for three times. The organic layer was dried with anhydrous MgSO_4_, and the solvent was evaporated under vacuum. The final product, azide-functionalized *β*-CD-PS-*b*-PtBA (i.e., *β*-CD-PS-*b*-P*t*BA-N_3_) was collected and dried at 40 ℃ in vacuum oven for 4 h. Alkyne-terminated mPEO (i.e., mPEO-propargyl) was obtained by nucleophilic substitution of the hydroxyl group on mPEO into the alkyne group. Briefly, mPEO-OH (5.0 g, 1.0 mmol) and THF (60 mL) was added in a 250 mL dried ampule. The system was then purged with argon. Then, The NaH (0.12 g, 5.0 mmol) was introduced under argon atmosphere. After the ampoule was placed in ice bath, propargyl bromide (5.0 mmol) was added dropwise during 2 h, and the reaction continued for 24 h at room temperature. The mPEO-alkyne was obtained by separation of the formed salts and precipitation in diethyl ether twice, and dried under vacuum at 40 ℃. Then, *β*-CD-PS-*b*-P*t*BA-N_3_ and mPEO-alkyne were dissolved in DMF (10 mL) in a dry ampule. CuBr and PMDETA were added, and the reaction mixture (mPEO-alkyne: *β*-CD-PS-*b*-P*t*BA-N_3_: copper bromide: PMDETA = 1.5 :1 :10 :10; molar ratio) was degassed by three freeze-pump-thaw cycles in liquid N_2_. The ampoule was first immersed in an oil bath at 90 ℃ for 24 h, and then taken out of the oil bath and placed in liquid N_2_ to terminate the polymerization. The mixture was diluted with THF and passed through the alumina column to remove the copper salt. The product was precipitated in cold methanol and dried in vacuum oven at 40 ℃ for 4 h, yielding 21-arm triblock copolymer, denoted as *β*-CD-PS-*b*-P*t*BA-*b*-PEO.

**(e)** ***Formation of β-CD*-*PS-b-PAA-b-PEO by hydrolysis of tert-butyl ester groups of PtBA block in β-CD-PS-b-PtBA-b-PEO***. Briefly, *β*-CD-PS-*b*-P*t*BA-*b*-PEO (0.3 g) was dissolved in 30 mL CH_2_Cl_2_, and 10 mL Trifluoroacetic acid (TFA) was then added. The reaction mixture was stirred at room temperature for 24 h. After the hydrolysis, the resulting *β*-CD-PS-*b*-PAA-*b*-PEO triblock copolymer was gradually precipitated in CH_2_Cl_2_. The final product was purified, washed with CH_2_Cl_2_, and thoroughly dried under vacuum at 40°C overnight to yield *β*-CD-PS-*b*-PAA-*b*-PEO. [2]

**Preparation of mesoporous TiO_2_ NFs**

The template used is the synthetic *β*-CD-PS-*b*-PAA-*b*-PEO (molecular weight (Mn) derived from the gel permeation chromatography result was ~439000 g·mol^-1^, and the polydispersity index (PDI) =1.33). In a typical synthesis of m-TNF, *β*-CD-PS-*b*-PAA-*b*-PEO (20 mg) was first dissolved in DMF (1.0 g) to form a clear solution in a glass vial, and then add 0.3g HCl dropwise to form the solution A. Polyvinylpyrrolidone (PVP, 0.2g) was dissolved in a mixed solvent (1g N, N-dimethylformamide (DMF) and 1 g absolute ethanol), forming the solution B. Then, titanium butoxide (TBOT, 1.2 g) was added to acetic acid (1.8 g), forming the solution C. The solution A and B were first mixed with stirring at room temperature for 30 min. The solution C was then added to the mixed solution and stirred at room temperature for 1 h. The above precursor was electronspun from a 5 mL syringe at a feeding rate of 0.5 mL/h by a syringe pump upon applying a 20 kV voltage. The distance between the needle tip and the collector was about 20 cm. Then, the as-spun fibers were thermally treated at 70 ℃ for 24 h to solidify the framework. Then, the fibers were first annealed at 350 ℃ under N_2_ atmosphere for 2 h to stabilize the mesoporous structure. Finally, the m-TNF were obtained by calcining at 500 ℃ in air for 2 h to remove the organic template and improve crystallinity.

The above electrospinning strategy is also applicable to the fabrication of mesoporous WO_3_, CeO_2_, ZrO_2_ and their composite NFs. Only need to replace the titanium butoxide with tungsten hexachloride, cerium nitrate, zirconium (IV) chloride and their mixture, respectively. The TiO_2_-based composite NFs and nanoparticle-decorated TiO_2_ NFs were prepared by adding a certain amount of other metal salts in the precursor solution, followed by the same electrospinning condition with that for m-TNF.

**Preparation of Pt-decorated mesoporous TiO_2_ NFs**

Preparation of m-TNF/Pt was via photodeposition method. In a typical preparation, 0.1 g m-TNF was dispersed in 20 mL solution (5 vol% ethylene glycol) containing H_2_PtCl_6_·6H_2_O (0.3 wt% Pt vs. TiO_2_) and kept stirring for 1 h in darkness to make the PtCl_6_ ions preferentially adsorbed on TiO_2_ followed by UV–vis irradiation (300 W xenon lamp) for 1 h to reduce the PtCl_6_ to Pt nanoparticles. After the photodeposition process, the grey m-TNF/Pt product was collected by centrifugation and washed with deionized water to remove ionic impurities until the ionic concentration below 1.0 ppm. Finally, the m-TNF/Pt were gained by drying at 60 ℃ overnight.

**Photocatalytic activity measurement**

The photocatalytic activities of the calcined m-TNF and m-TNF/Pt were evaluated by tracing the concentrations of tetracycline (TC) as a function of irradiation time under visible light irradiation. 20 mg of the prepared catalyst was added to 100 mL of TC aqueous solution (20 mg/L) in a quartz beaker. Prior to irradiation, the mixture was dispersed in an ultrasonic bath for 10 min and stirred for 90 min in dark to establish an adsorption-desorption equilibrium. During irradiation, a xenon lamp (Model CEL-HXF 300 W, Global Xenon Lamp Power) was used as light sources to trigger the photocatalytic reaction and about 3 mL of the suspension was withdrawn at 20 min intervals to record the dynamic TC concentration using a UV–vis spectrophotometer (Shimadzu, Model UV-3600).

**Characterization**

Scanning electron microscopy (SEM) images were obtained on a TESCAN MAIA3 field-emission scanning electron microscope. Transmission electron microscopy (TEM) images were taken at a JEM-2100F with an accelerating voltage of 200 kV. A Rigaku D/Max-2550 PC diffractometer (Tokyo, Japan) was used to collect the X-ray diffraction (XRD) data at a 2θ range from 10° to 90°. The N_2_ adsorption-desorption isotherms were measured by Quantachrome Autosorb-IQ instrument. The specific surface area was calculated based on the Brunauer-Emmett-Teller (BET) method using the adsorption data in a relative pressure (P/P_0_) ranged from 0.005 to 0.25. The pore size distribution and pore volume were calculated from the adsorption branches of isotherms using the Barrett-Joyner-Halenda (BJH) method. The total pore volume was estimated from the adsorbed amount at a P/P_0_ of 0.995. X-ray photoelectron spectrum (XPS) was recorded by Escalab 250xi X-ray photoelectron spectrometer. All the binding energies were modified using the C 1s standard peak at 284.6 eV.  ^1^H-NMR spectra were recorded on an AVANCE NEO 400 MHz full digital NMR spectrometer with tetramethylsilane as the internal standard and CDCl_3_ as the solvent. Photoluminescence emission spectra were characterized by fluorescence spectrophotometer (FLS1000). UV–visible spectrophotometer (TU-1901) was applied to evaluate the optical properties. Electron spin-resonance spectroscopy (ESR) measurements at X-band were carried out on a Bruker EMXPLUS spectrometer. The gel permeation chromatography (GPC) result was obtained on a BI-MWA type chromatographer with a refractive index detector using DMAc as the mobile phase. Characterizations of unimolecular micelles of amphiphilic multi-arm block copolymers by dynamic light scattering (DLS).

| 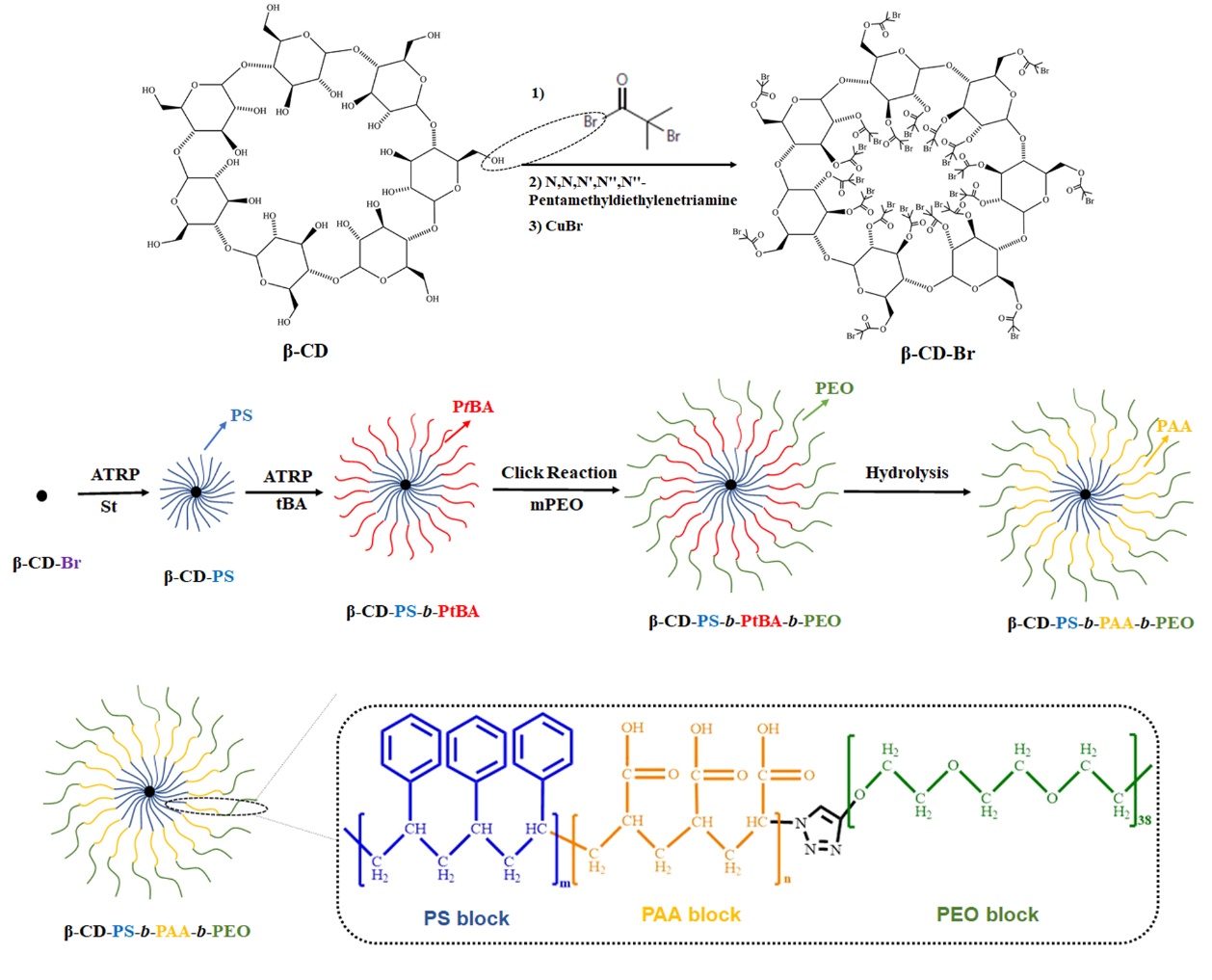 |
| --- |
| Figure S1. Schematic representation of the synthetic route of *β*-CD-PS-*b*-PAA-*b*-PEO template. |

| 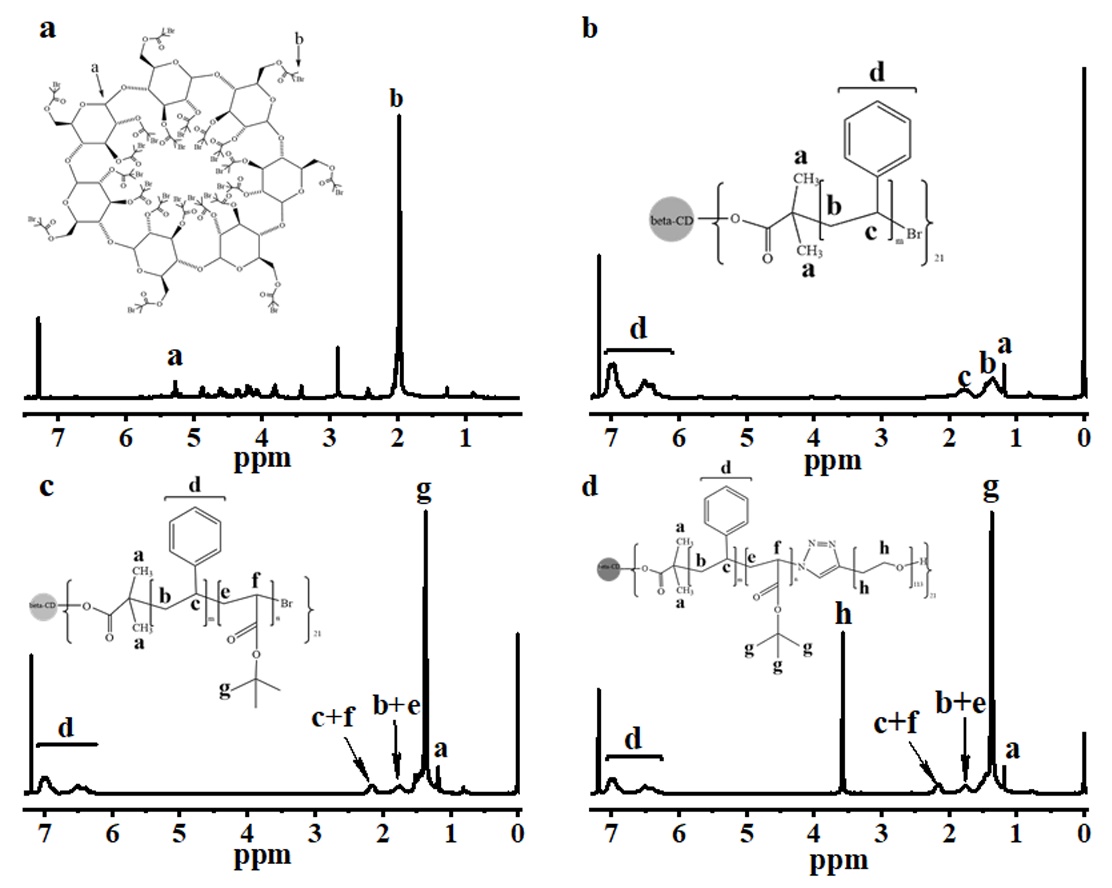 |
| --- |
| Figure S2. ^1^H NMR spectrum of 21Br-*β*-CD (a), *β*-CD-PS (b), *β*-CD-PS-*b*-P*t*BA (c), *β*-CD-PS-*b*-P*t*BA-*b*-PEO (d), solvent: CDCl_3_. |

| 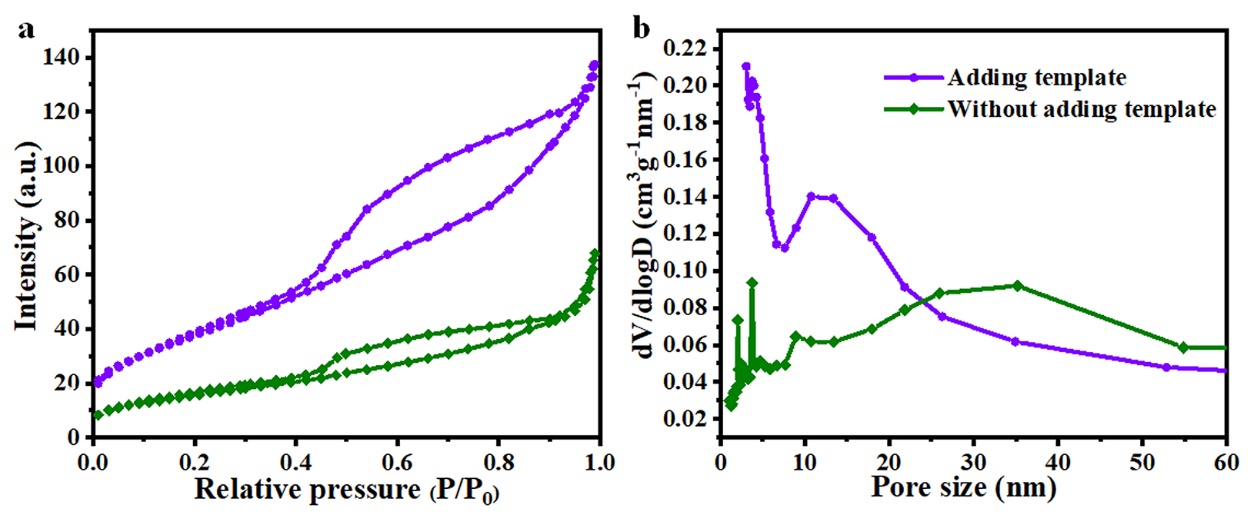 |
| --- |
| Figure S3. Nitrogen sorption and desorption isothermals (a) and pore size distribution (b) of the NFs prepared without *β*-CD-PS-*b*-PAA-*b*-PEO and with *β*-CD-PS-*b*-PAA-*b*-PEO. |

| 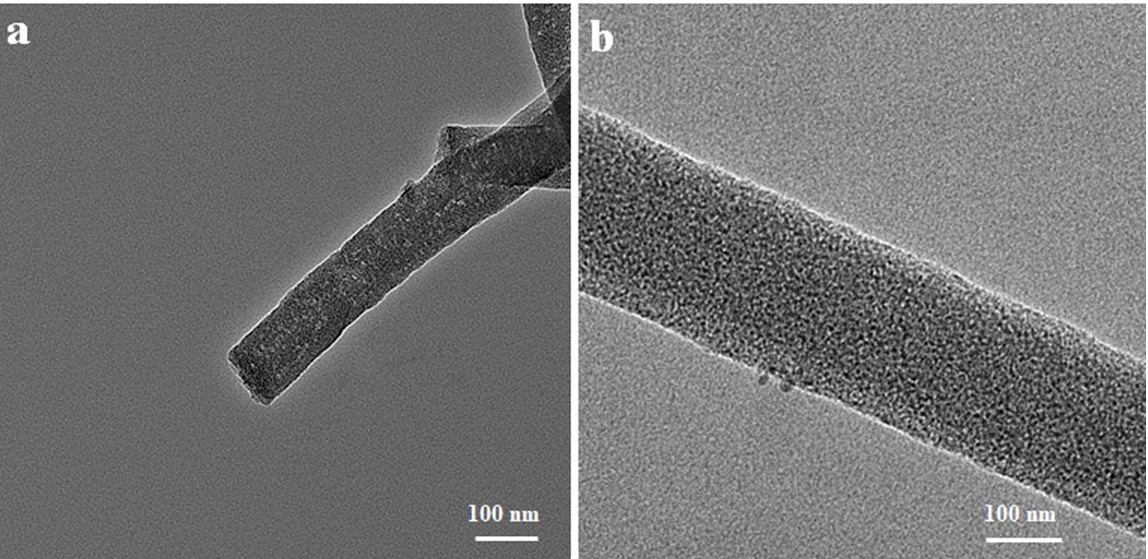 |
| --- |
| Figure S4. TEM images of the NFs prepared with β-CD-PS-*b*-PAA-*b*-PEO (a) and without β-CD-PS-*b*-PAA-*b*-PEO (b). |

| 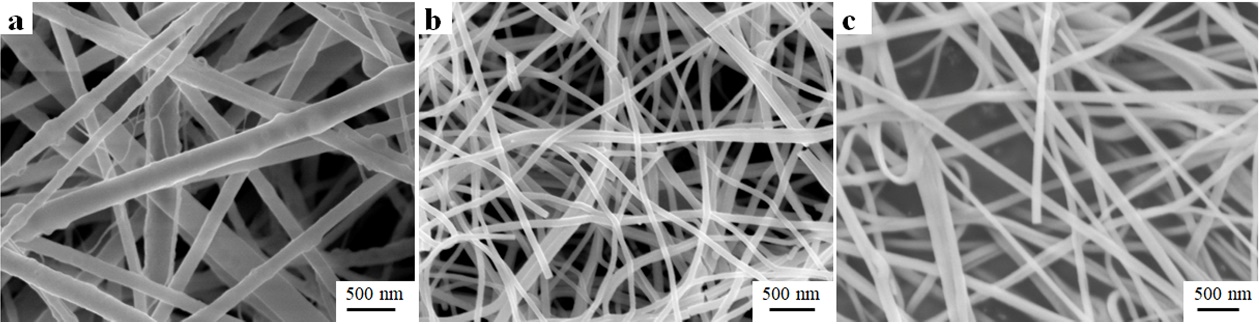 |
| --- |
| Fig. S5 SEM images of the mesoporous WO_3_ (a), ZrO_2_ (b) and CeO_2_ (c) NFs. |

| 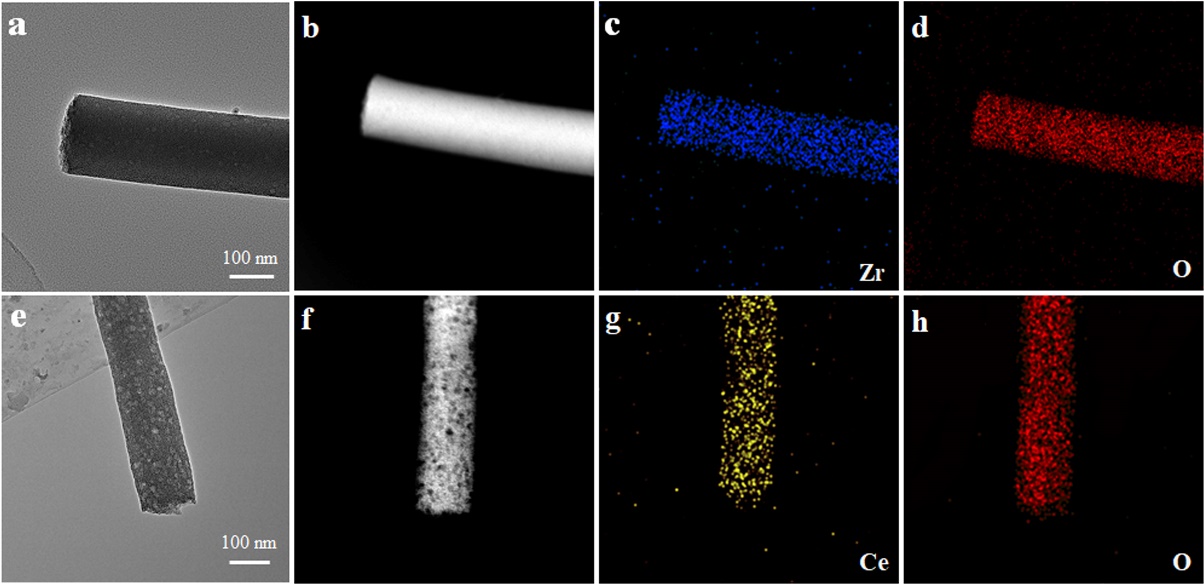 |
| --- |
| Fig. S6 TEM (a) and STEM (b) image of mesoporous ZrO_2_ NFs and the EDX maps of Zr (c) and O (d). TEM (e) and STEM (f) image of mesoporous CeO_2_ NFs and the EDX maps of Ce (g) and O (h). |

| 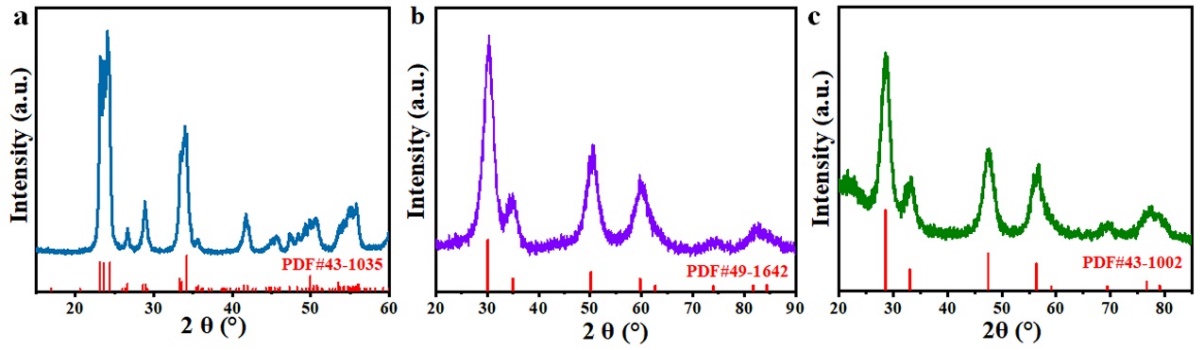 |
| --- |
| Fig. S7 XRD patterns of the mesoporous WO_3_ (d), ZrO_2_ (e) and CeO_2_ (f) NFs. |

| 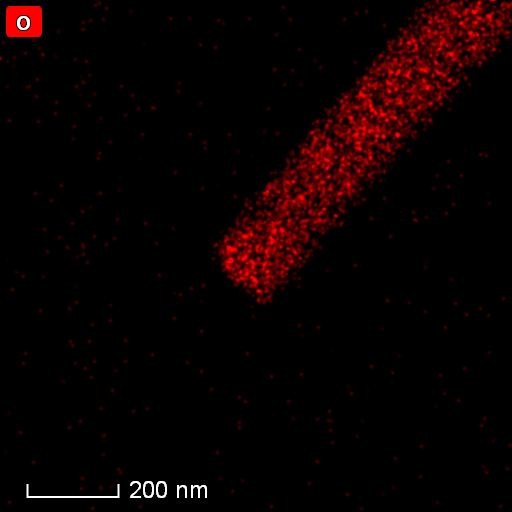 |
| --- |
| Fig. S8 The EDX maps of O on the mesoporous TiO_2_/WO_3_ NFs. |

| 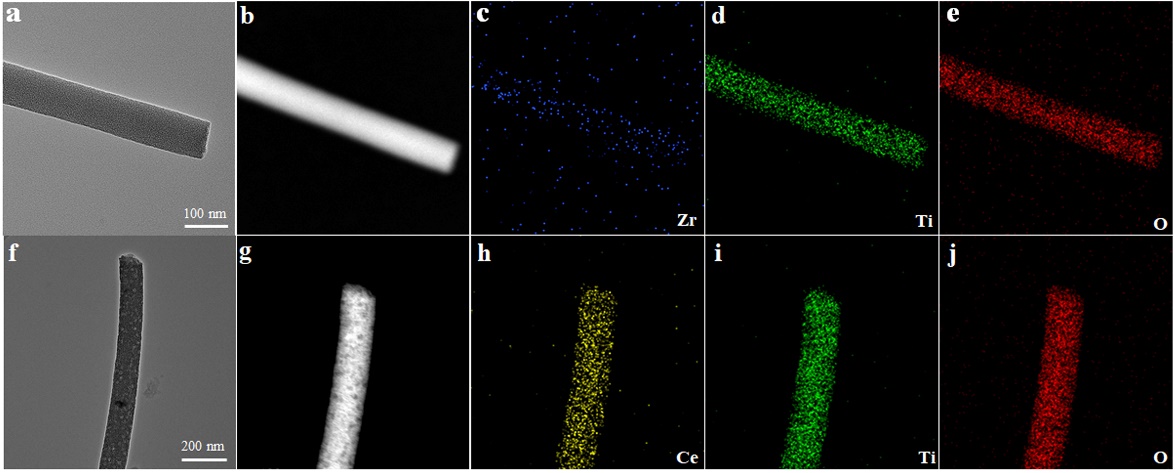 |
| --- |
| Fig. S9 TEM (a) and STEM (b) image of mesoporous TiO_2_/ZrO_2_ NFs and the EDX maps of Zr (c), Ti (d) and O (e). TEM (f) and STEM (g) image of mesoporous TiO_2_/CeO_2_ NFs and the EDX maps of Ce (h), Ti (i) and O (j). |

| 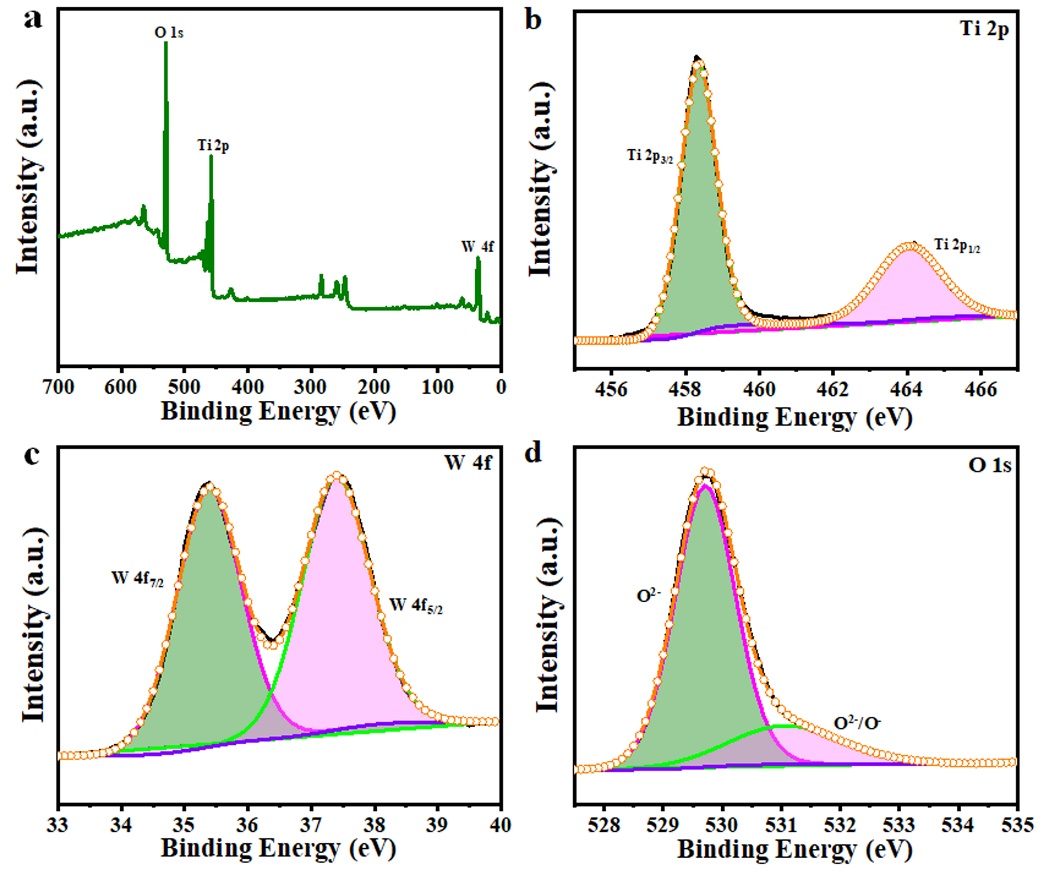 |
| --- |
| Figure S10. XPS spectra (a) of mesoporous TiO_2_/WO_3_ NFs. High-resolution Ti 2p (b), W 4f and O 1s (d) XPS spectra of mesoporous TiO_2_/WO_3_ NFs. |

| 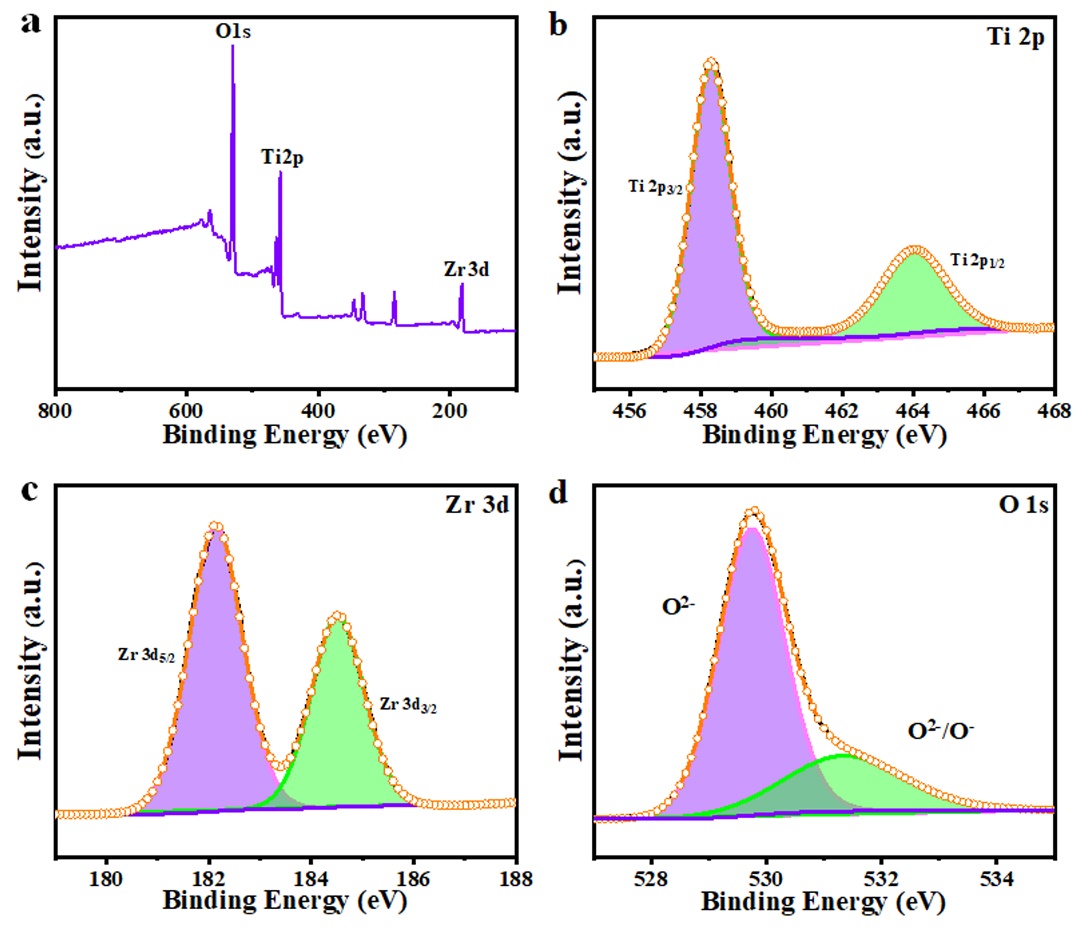 |
| --- |
| Figure S11. XPS spectra (a) of mesoporous TiO_2_/ZrO_2_ NFs. High-resolution Ti 2p (b), Zr 3d and O 1s (d) XPS spectra of mesoporous TiO_2_/ZrO_2_ NFs. |

| 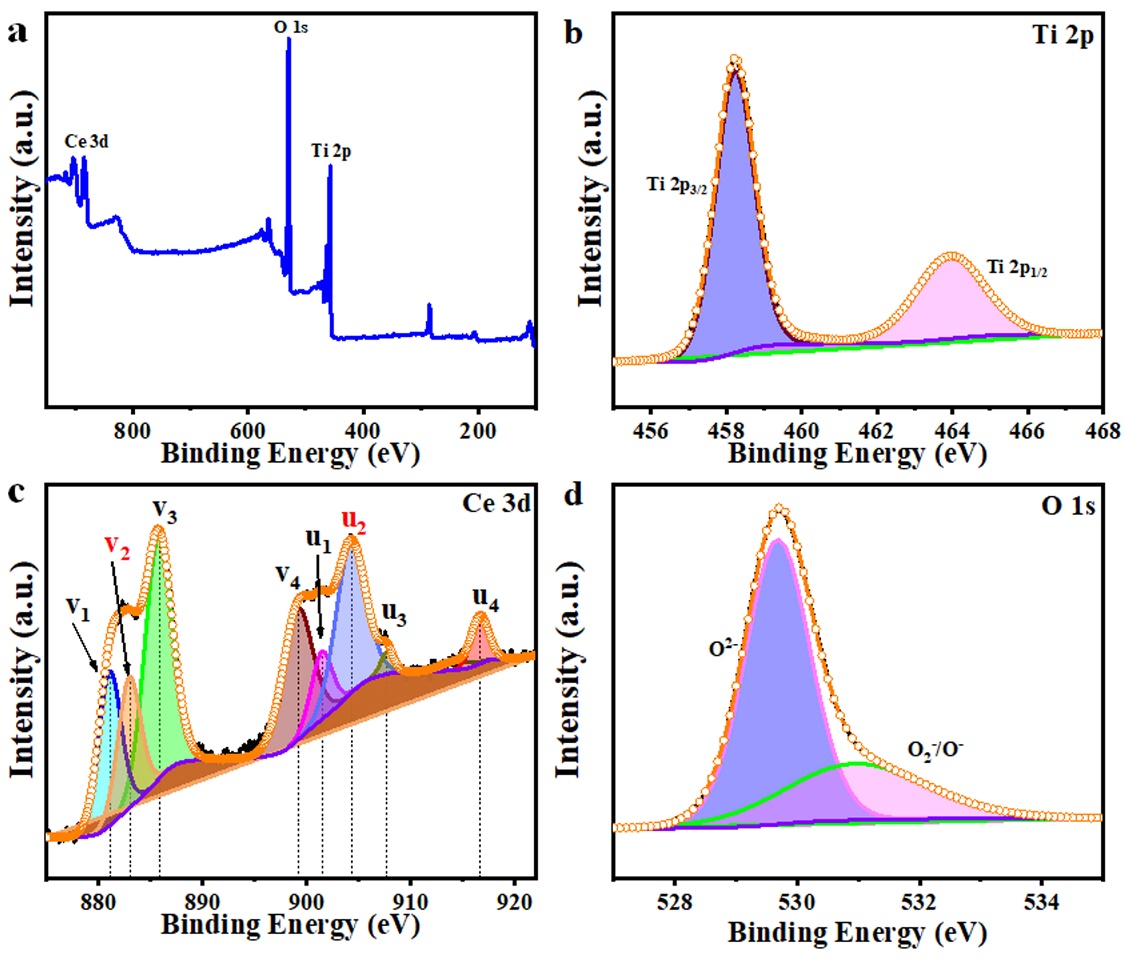 |
| --- |
| Figure S12. XPS spectra (a) of mesoporous TiO_2_/CeO_2_ NFs. High-resolution Ti 2p (b), Ce 3d and O 1s (d) XPS spectra of mesoporous TiO_2_/CeO_2_ NFs. |

| 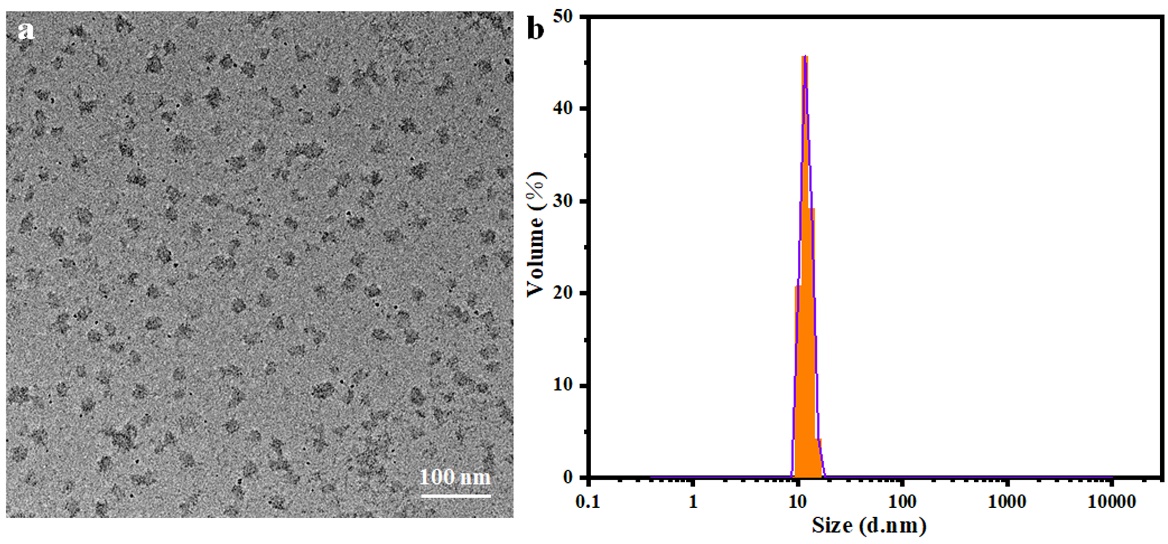 |
| --- |
| Figure S13. TEM images of the *β*-CD-PS-*b*-PAA-*b*-PEO dissolved in DMF (a). DLS measurement on unimolecular micelles of *β*-CD-PS-*b*-PAA-*b*-PEO in DMF (b). |

|  |
| --- |

Reference

[1] X. Pang, L. Zhao, M. Akinc, et al. Novel amphiphilic multi-arm, star-like block copolymers as unimolecular micelles. Macromolecules. 44. 10. (2011) 3746-3752.

[2] X. C. Pang, L. Zhao, W. Han, et al. A general and robust strategy for the synthesis of nearly monodisperse colloidal nanocrystals. Nat. Nanotechnol. 8. (2013) 426-431.
